# Listening to diverse community voices: the tensions of responding to community expectations in developing a male circumcision program for HIV prevention in Papua New Guinea

**DOI:** 10.1186/1471-2458-13-749

**Published:** 2013-08-13

**Authors:** Anna Tynan, Peter S Hill, Angela Kelly, Martha Kupul, Herick Aeno, Richard Naketrumb, Peter Siba, John Kaldor, Andrew Vallely

**Affiliations:** 1 Australian Centre for International & Tropical Health, School of Population Health, University of Queensland Herston Road, Herston, 4006 Queensland, Australia; 2 Sexual & Reproductive Health Unit, Papua New Guinea Institute of Medical Research (PNG IMR), Eastern Highlands Province 441, P.O. Box 60, Goroka, Papua New Guinea; 3 International HIV Research Group, School of Public Health and Community Medicine, University of New South Wales, Sydney, Australia; 4 Public Health Interventions Research Group, The Kirby Institute, University of New South Wales, 45 Beach Street Coogee, 2034 New South Wales, Australia

**Keywords:** Community participation, Male circumcision, Penile cutting, HIV

## Abstract

**Background:**

The success of health programs is influenced not only by their acceptability but also their ability to meet and respond to community expectations of service delivery. The World Health Organization (WHO) and the Joint United Nations Programme on HIV/AIDS (UNAIDS) have recommended medical male circumcision (MC) as an essential component of comprehensive HIV prevention programs in high burden settings. This study investigated community-level perceptions of MC for HIV prevention in Papua New Guinea (PNG), a setting where diverse traditional and contemporary forms of penile foreskin cutting practices have been described.

**Methods:**

A multi-method qualitative study was undertaken in four provinces in two stages from 2009 to 2011. A total of 82 in-depth interviews, and 45 focus group discussions were completed during Stage 1. Stage 2 incorporated eight participatory workshops that were an integral part of the research dissemination process to communities. The workshops also provided opportunity to review key themes and consolidate earlier findings as part of the research process. Qualitative data analysis used a grounded theory approach and was facilitated using qualitative data management software.

**Results:**

A number of diverse considerations for the delivery of MC for HIV prevention in PNG were described, with conflicting views both between and within communities. Key issues included: location of the service, service provider, age eligibility, type of cut, community awareness and potential shame amongst youth. Key to developing appropriate health service delivery models was an appreciation of the differences in expectations and traditions of unique cultural groups in PNG. Establishing strong community coalitions, raising awareness and building trust were seen as integral to success.

**Conclusions:**

Difficulties exist in the implementation of new programs in a pluralistic society such as PNG, particularly if tensions arise between biomedical knowledge and medico-legal requirements, compared to existing socio-cultural interests. Community participatory approaches offer important opportunities to explore and design culturally safe, specific and accessible programs.

## Background

Engaging communities in health programs is influenced by the type of intervention that is going to be rolled out and the interest, aversion or beliefs that a community may already have [1,2]. Male circumcision and other penile cutting practices have historically been a part of many different ethnic, religious and traditional cultures around the world with considerable variability of age at which circumcision takes place, tradition of the ceremony, and type of cut [3-9]. Following the recommendation by the World Health Organization (WHO) and the Joint United Nations Programme on HIV/AIDS (UNAIDS) in 2007 that medical male circumcision (MC) be considered an essential component of comprehensive HIV prevention programs in high burden settings, a number of African countries have embarked on ambitious MC scale-up programs. Many of these high burden countries however, continue to document slow rates of roll out and uptake [10,11]. Although the acceptability of MC programs has been cited as a key consideration in determining program appropriateness in different contexts [12,13], further understanding of how to engage communities and facilitate uptake following confirmation of acceptability is integral for ongoing success of any public health program [7,14-16]. In Papua New Guinea (PNG), a country where a significant number of diverse penile cutting practices have been described to be already apart of the socio-cultural fabric of many communities, interest in aligning MC with other HIV intervention programs has been raised [17-20]. This study is an exploratory study using interviews and participatory tools to investigate the potentials and challenges of integrating community participation into health service delivery. This research was conducted as part of a multi-disciplinary, community-based research program to investigate the acceptability, epidemiological impact, cost-effectiveness and options for program implementation of MC for HIV prevention in PNG.

### Papua New Guinea and male circumcision

Papua New Guinea has a population that is geographically and culturally diverse with over 700 disparate cultural group and 832 distinctive, mutually unintelligible languages [21-25]. In many parts of PNG, particularly in urban settings and regions of high HIV transmission, populations are extremely diverse with individuals often identifying within close family groupings to traditions and language, even when they have gravitated to urban settlements [22,26]. Recent collaborative research on MC in PNG has indicated that MC would be notionally acceptable and could have an impact on the national epidemic [18,27]. However concern also exists over health system capacity to deliver such a program, based on the challenges faced by the National Department of Health and partners in delivering even basic health services due to the geographical isolation of populations; limited road infrastructure and rugged terrain; limited and inconsistent funding availability; difficulties establishing, and maintaining appropriate health information systems to monitor service coverage and quality; and diminishing quality of services once established, particularly in rural areas [28-31]. Despite this and no formal national policy, a MC program for HIV prevention has already been established in East Sepik Province (ESP) and has shown varying degrees of success [31].

The tremendous diversity of socio-cultural contexts, health beliefs and practices in PNG, influences how the objectives and potential benefits of biomedical interventions for HIV prevention are perceived and understood at local level [32]. For example, penile cutting practises in PNG are embedded in customary rituals and contemporary practices (influenced by peers and the socio-cultural environment) [17-20]. These practices also encompass various forms of penile cutting that do not commonly involve circumferential cutting or full excision of the foreskin as currently indicated for HIV prevention [9,17,19,20,33,34]. With such tremendous geographical constraints and significant linguistic and cultural diversity, a clear understanding of not only the acceptability of new health programs in different communities, but also consideration of how to involve these communities in meaningful and sustainable ways alongside the goals of the health program in the face of political, economic, cultural and epidemiological pressures and health system capacity is warranted [35,36].

### Community engagement

A community-centred approach to health care delivery involves a balanced consideration of the rights, needs, responsibilities and capacities of all the constituents and stakeholders of a health system with an underlying value of social justice and a focus on improving local communities’ self-reliance and participatory decision-making [37]. Inspired by the original Alma Ata declaration of 1978, community participation is considered one of the pre-requisites for the success of the primary health care movement, and a variety of international and national-level policy documents still cite the importance of engendering this approach [38,39]. The World Health Report 2008 "Primary Health Care: Now more than ever" has more recently re-ignited the importance of organizing health services around people's needs and expectations [40]. However, employing community participation strategies can be challenging and there exists a range of obstacles in the health sector due to competing social, political and economic interests [41,42].

Key obstacles to meaningful community participation in health service delivery include difficulties defining what community participation should involve and how it should work [2,39,42-44]; the potential to use the rhetoric of community participation for the legitimacy of political regimes or international organisational interests [41,45,46]; power, resource and knowledge asymmetries between policy makers, health staff and communities [2,47]; inadequate health infrastructures and financial resources to support a community participation programme [2]; inadequate understanding of conflicts of interest, opposing political ideologies and group rivalries characterizing some communities [2,48]; and the impact that community history, culture and socio-political development has on the community-health service interface and the ability to build trustful relationships in these contexts [39,49-51]. Complications also exist in recognising the extent to which views are held by different community members following failures to identify key community representatives or stakeholders [47]. However, one of the most consistent difficulties to community collaboration in public health programs is the lack of accepted definitions of ‘community’ and ‘participation’ [39,42,52].

Standardization of the concept of community has been argued to be neither possible nor desirable due to the complexities involved in defining the community and the substantial variability of circumstances and socio-political environments [39]. Communities are an aspect of collective and individual identity which are the result of a web of relations based on socio-cultural, historical, ideological and economic characteristics and not necessarily geographically defined, particularly where there are imbalances in resource availability, cultural heterogeneity, ethnic tensions, or itinerant populations [42-46,48,53,54]. Communities incorporate a complex affiliation of people who do not always share a common identity, with many different and overlapping identities within its boundaries that vary over time [2,42]. These identities may be based on, age; gender; engagement in joint action; ethnicity; social ties, shared experiences; perspectives, expectations, and interests; and socio-economic status [47,55-57].

Community participation may be considered on a continuum from the community merely engaging in health programs, to participating directly in the development and maintenance of a program, to complete empowerment and ownership of health programs [39,58,59]. Successful community participation should be viewed as a process and not an intervention and, it has been advocated that communities should be given the opportunity to define their idea of ‘participation’ [2,48,49]. This definition, must also be considered within the influence of political philosophies, community and stakeholder perceptions of existing and expected levels of participation, community priorities and interests, the acceptability of the implementation of participatory interventions and health program expectations [2,39,54]. Community participation programmes should therefore remain dynamic and responsive to changing needs [2,22,60].

The importance of understanding communities and their relationships to the health system and the health program that is being delivered should not be underestimated and can play an essential role in delivering services. In addition to community acceptability, understanding community-level meanings and potential attitudes and inclinations towards different service delivery models is essential, particularly when evidence of similar pre-existing practices already exist within the socio-cultural environment. The aim of our study was therefore to explore community opinions regarding service delivery models for the implementation of MC for HIV prevention in PNG and how participatory methods may assist.

## Methods

### Study design

A multi-method qualitative study was undertaken in four provinces in PNG: National Capital District (NCD); Eastern Highlands Province (EHP); East Sepik Province (ESP); and West New Britain Province (WNBP). The research was conducted in two stages from 2009 until 2011. In each of the study sites, focus group discussions (FGDs) and in-depth interviews (IDIs) were conducted during stage 1; and participatory community workshops conducted in stage 2.

### Study sites

**Figure 1:**
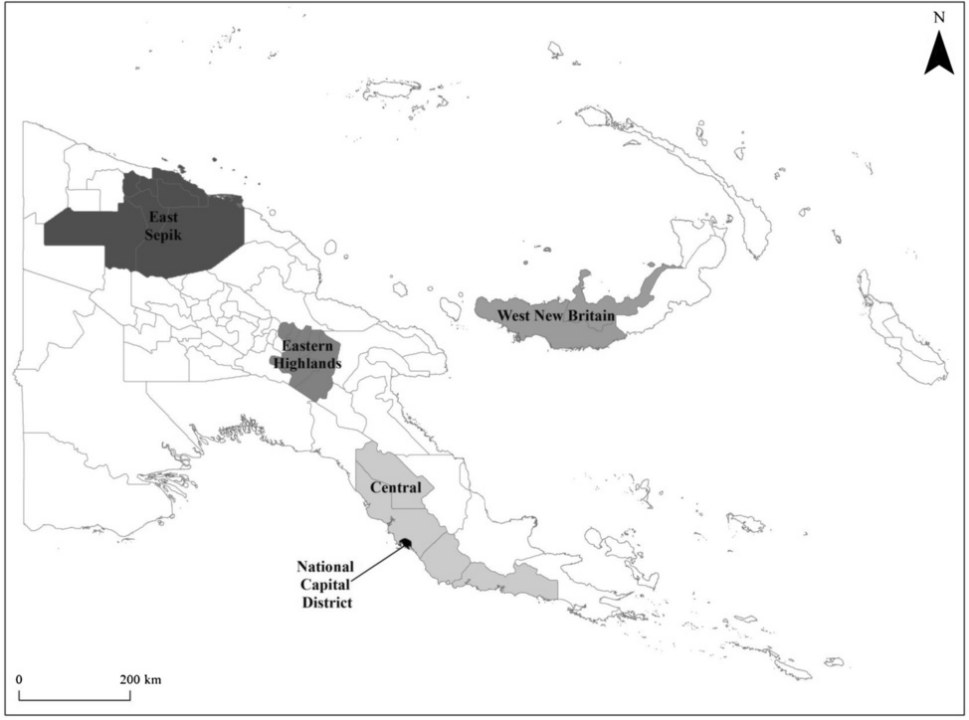
Map of Papua New Guinea with study provinces highlighted.

Study sites were selected during a National Stakeholders Workshop held in Port Moresby in May 2008 based on representation of socially and geographically diverse regions of the country (Figure 1). Approval from key local authorities, chiefs and other stakeholders were obtained prior to the arrival of the research team in each study location. In each province, the research team conducted research amongst multiple cultural and language groups highlighting the complexity of pluralistic communities in each setting.

Port Moresby, NCD was selected because it is the national capital with a diversity of people coming from all regions of the country. Informal urban settlements are a feature of Port Moresby and have been defined as groups of households in localities and in conditions that contravene the laws and regulations of the state [61]. These settlements are characterised by diverse cultural groups, haphazard housing, poor access to basic amenities, and at least some temporary housing [61,62].

Eastern Highlands Province (EHP) is considered a traditionally non-penile cutting cultural area. It is linked to other areas of the country through the Highlands Highway, which serves as one of the country’s primary economic transport routes, linking coastal ports in Morobe and Madang Provinces with major towns and other destinations in the Highlands. The highway is the main conduit for people travelling and migrating from different areas of PNG. It has also been considered a potential channel for the transmission of STIs and HIV between provinces that are serviced by the road [63].

The Sepik river basin (which includes East Sepik Province) is linguistically the most complex area in PNG with about 200 spoken languages [64]. Research was undertaken in a number of sites within East Sepik Province (ESP) including in the provincial capital and several regional centers surrounding the capital (including Angoram and Maprik). East Sepik Province has a history of traditional penile cutting practices, although recent community studies suggest that these practices have been discontinued in many areas [17,65,66]. Currently there is a medical MC program for HIV prevention operating in ESP since 2006 that also includes community outreach.

West New Britain Province (WNBP) also has a history of traditional penile cutting practices [65-69], and recent research indicates that these practices continue in some communities [17,18].

### Study participants

In order to ensure effective representation and to avoid misunderstandings and distrust developing between potential participants and external researchers, extensive community consultation was carried out with key stakeholders and the findings triangulated with previous research on penile cutting practices in PNG [57]. Community consultation and reflection also allowed for insight into existence of community definitions relevant to the research question including those who engaged in penile cutting practices, age definition (youth were typically considered unmarried or <25years), gender perceptions, and differing community histories. An iterative, purposive sampling technique was used to identify potential study participants following initial contact and interviews with key local stakeholders and community members at each study location in both stage 1 and 2.

### Data collection

In stage 1, a total of 272 men and 210 women (N=482) participated in 82 IDIs, and 45 FGDs that were conducted by experienced locally-based fieldworkers trained in qualitative data collection methods and fluent in both *Tok Pisin* (a lingua franca of PNG) and English in the four study locations (Table 1). The data collection processes have previously been described [17,18,70]. In brief, interview guides were developed that enabled interviewers to explore perceptions and preferences for service delivery, should the Government of PNG decide to go ahead in implementing a MC program for HIV prevention.

**Table 1:** Summary of interviews, focus group discussions and workshops conducted


**Stage 1**					
	**ESP**	**EHP**	**WNBP**	**NCD**	**Total**
Women					
* Focus group discussions*	7	4	5	5	**21**
* In-depth interviews*	3	2	3	10	**18**
Men					
* Focus group discussions*	4	5	8	8	**25**
* In-depth interviews*	16	7	3	10	**36**
**Stage 2**					
	**ESP**	**EHP**	**WNBP**	**NCD**	**Total**
Community women workshop (older)	-	1	-	-	1
Community women workshop (youth)	1	1	-	-	2
Community men workshop	1	1	-	-	2
Community leader workshop (lay)*	-	1	-	-	1
Community leader workshop (professional)**	1	1	-	-	2
Total number of workshops					**8**

*Other general community leaders including youth leaders, women’s group leaders.

** Professional staff from health facilities, department of health or other related public services.

**Figure 2:**
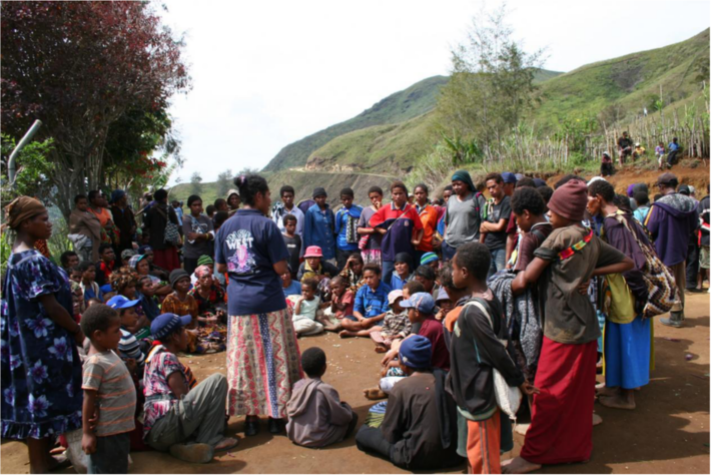
Participatory workshop on service delivery models for MC for HIV prevention.

**Figure 3:**
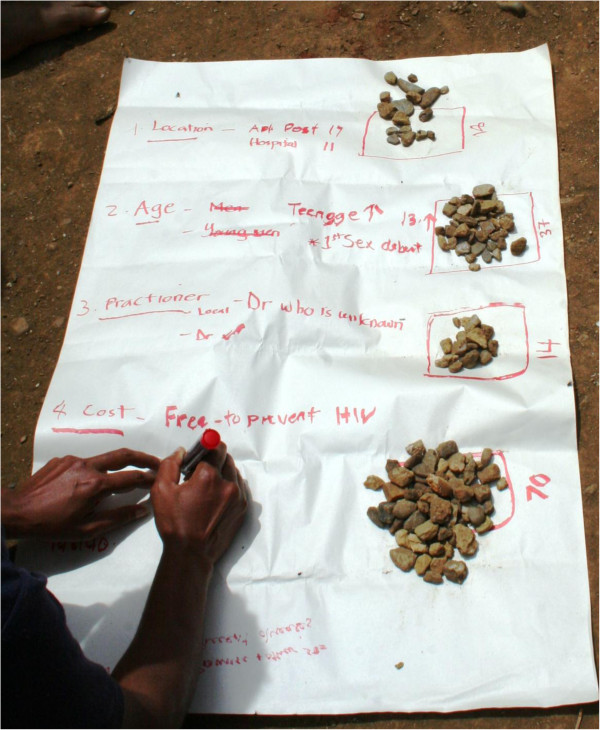
Example of an activity completed during participatory workshops.

In Stage 2, eight community feedback workshops were conducted in areas previously visited during Stage 1, to review key themes, consolidate earlier findings and assist with dissemination of research back to study communities (Figure 2). Due to logistical and security reasons, community workshops were conducted in EHP and ESP only, with other channels of dissemination being used in WNB and NCD e.g. media releases, stakeholder workshops, and presentations at annual medical symposia of the PNG Medical Society, and at the National Policy Forum on Male Circumcision for HIV Prevention, held in Port Moresby in November 2011 [71,72]. Five workshops were completed with community members and three workshops completed with community leaders and local health care professionals. Community workshops utilised a variety of participatory research tools successfully employed by the research team in earlier studies [57,73,74], such as listing, scoring and ranking to highlight community-level priorities for future service delivery. Participants were asked to list considerations for implementing a MC program in their communities and to rank the issues from least to most important. Ranking was completed by either voting with stones or in-depth discussions until consensus was obtained (Figure 3). In the workshop conducted with community leaders in ESP, the group chose to consider the current MC program and to list facilitators and barriers to current service delivery.

### Data analysis

All FGDs and interviews in Stage 1 were digitally recorded, transcribed verbatim and where necessary translated from *Tok Pisin* to English, before undergoing thematic analysis which was carried out by five researchers based at the PNG Institute of Medical Research (IMR) in Goroka. Accuracy and equivalency of translations were cross checked by two bilingual translators. Any incongruities were then negotiated between the two bilingual translators. Thematic analysis was carried out at the end of stage 1 and stage 2. In stage 1 a codebook was developed and all interviews were double coded. In cases of discrepancy in coding a third researcher coded the selected text in question. Themes identified in Stage 1 provided the grounding for structuring the workshops in Stage 2. In Stage 2, qualitative analysis of posters generated from workshop activities along with field notes written during participatory workshops was conducted and organised into themes. Finally, all data was subjected to additional thematic analysis that was facilitated by qualitative data management software (NVivo 8; QSR International Pty Ltd, Australia). Qualitative data analysis focused on comparing and contrasting specific themes as they occurred including the barriers to service delivery, preference to certain attributes of the service delivery models, and insights into unique community perceptions.

### Ethical considerations

Ethical approval was granted in PNG by the Institutional Review Board of the PNG Institute of Medical Research, the National AIDS Council Research Advisory Committee and the Medical Research Advisory Committee of the National Department of Health. In Australia, ethics approval was provided by the Human Research Ethics Committees of the University of New South Wales and the University of Queensland. The purpose of FGDs, IDIs and participatory workshops were explained to study participants by a trained field worker. All those who participated in an FGD or IDI provided written informed consent prior to the start of the interview (participants who were unable to read or write provided a witnessed thumbprint). For those taking part in community workshops, verbal consent was obtained prior to commencing the activity. This research adheres to the qualitative research review guidelines (RATS).

## Results

There was considerable diversity in community opinions of how a future MC program for HIV prevention should be implemented in PNG. Clear distinction of program design requirements existed between traditional penile cutting communities and non-traditional penile cutting communities. However, a number of often conflicting suggestions were offered within communities with regard to service location (community-based *vs.* hospital based delivery); people providing the service (traditional and/or local (contemporary) cutters *vs.* trained health workers); cost (free *vs.* fee-based service); and target population strategy (infants and children *vs.* young men or adults) (Table 2). These divergences in opinion were embedded in gender roles, socio-cultural contexts and division among age groups. While all participants highlighted the need to ensure implementation of a ‘safe’ procedure, detailing what this would entail, and how much health system involvement was needed varied.

**Table 2:** Preferred service delivery options for a male circumcision program for HIV prevention

			
**Traditional penile-cutting communities**			
**Location**		**Service provider**	
**Health facility**	**Traditional house**	**Health worker**	**Traditional cutter**
To increase safety and precision of cut	To preserve culture.	Health worker could be involved in traditional ceremony as a guest at traditional house	If provided with skills and resources from the government
Initial procedure completed at health facility but all follow up and medication completed in traditional ways	To strengthen the community	Health worker to complete in health facility	To increase ease of access for some communities
Completed at health facility but followed up with customary celebrations	For the community to have more control of program.		
For poor families who can’t afford customary celebrations.	Because it defined customary practice		
	Completed only at traditional house to respect custom		
	For wealthy families to promote social standing		
**Financing**		**Target population**	
**Free**	**Payment**		
Should be free if completed by government	For traditional purposes only	> 10 years as per traditional custom	
Should be free if completed by government	In kind or in gratitude, a part of cultural celebration	School age	
**Communities that do not engage in traditional penile cutting**			
**Location**		**Service provider**	
**Health facility**	**Community location**	**Health worker**	**Peer/non-health worker**
At health facility to increase safety of procedure	Complete in secret community location	Health worker to increase safety	Use of local cutter due to loss of potential income if Health worker only involved
At health facility but it would need to be discrete	For ease of access	Health worker from outside community to increase secrecy and safety	Accredited local cutter to alleviate human resource burden on health system
Not at major hospital as it is too public	Going to health facility can be expensive and time consuming	Male health worker as it would save embarrassment for patient and female health worker	
Community outreach program to aid post level and if possible village level to assist in mitigating access issues for rural people			
**Financing**		**Target population**	
**Free**	**Payment**	**< 5 years**	**>10 years**
Because the government was promoting it	To increase accountability for action	Because many children are exposed to sex early	Around time of sexual debut
A cost would be a barrier to service uptake	In kind or in gratitude	Other countries circumcise babies	Older because boys they would be stronger physically and mentally to handle pain and procedure
Young men would not have access to cash due to poor employment options			Older so that children would have a choice
			If too high then this may impact on uptake

### Diversity in communities where traditional forms of penile cutting are practiced

Interest in a medical MC program to be linked to traditional practices and completed alongside traditional ceremony in a “*haus man*” or “*haus boi*” (a house traditionally designated for the exclusive use of men/male youth and often the place where initiation takes place) was evident in ESP and WNBP communities. However, opinion on how this was to be incorporated differed, particularly between men and women in WNBP communities and male youth and other groups in ESP. These distinctions appeared to be influenced by the socio-cultural and political histories of the area.

#### East Sepik province

##### Who should provide the service and where should it be located?

East Sepik Province has a history of traditional penile cutting practices and a current medical MC program for HIV prevention operating from the main hospital in Wewak since 2006. Most participants agreed that the procedure should be completed by a health worker (HW) in a health facility, including men who had previous experience of penile cutting.

*I just heard it now that they cut it in the health facility and I feel that it’s more safe and good to go to the health facility to get cut. They will cut it properly, in the right way. The doctor can also check you properly and you can get good advice regarding your skin.* Man with penile alteration, In-depth Interview, ESP

Women also tended to agree that the procedure be completed in a health facility.

*I think that health facilities are better because they cut and treat it properly. To cut elsewhere or in the bush like custom ways is not good because they use bamboo and the same blades to cut - different diseases can be transmitted then.* Young women focus group discussion, ESP

However, for many men, incorporating a medical MC program within the ‘haus boi’ tradition would also be a way of maintaining the Sepik culture, a sentiment that was strongly held by local government stakeholders and community leaders.

*I will support it because the male children must stay in the ways of a male child. They will cut the skin, stay in the ‘haus boi’ and they will stay to strengthen the community*. Man, In-depth Interview ESP

During the workshops, male youth from settlement areas in Wewak suggested their preferred service model would be medical intervention for the initial cut, followed by customary practices and wound care (using traditional medicines) during the healing period (Table 3). Reasons provided for this preference was due to concerns about the costs involved in accessing health facilities and medicines. Other men suggested that the community have more control over the program with the health system assisting as they required. A few male youths in ESP advised they would prefer the training of traditional cutters to implement the program entirely.

*Yes if they* (the government) *can provide the materials to cut like blades, medicines, gauze and give some small guidelines to follow regarding health and safety, it will be alright for these doctors* (traditional cutters) *in the village to cut.* Young man, In-depth Interview, ESP

**Table 3:** Summary of workshops from community groups

		
**East Sepik Province workshops**		
**Community leaders workshop Wewak n= 5**		
**Review of current male circumcision program Advantages and Disadvantages**		
***Advantages***		***Disadvantages/challenges***
1 Maintains the Sepik culture: (particularly if done in traditional way)		Inadequate materials for the procedure so there is a tendency of reusing the same blades and materials if done in haus man
2 Influx of people for circumcision due to:		Scarce human resources
a Prevention of STI (HIV)		Shortage of testing materials
b Prevention of cervical cancer		Funds
c Reduction of STI/HIV cases		Lack of information
3 Entry point to VCT		Having multiple sexual partners post circumcision
4 Referrals made to access proper medical services		
5 Behavioral change due to increased involvement in VCT		
**Young women Wewak n=6**		
***Factor***	***Consensus vote***	***Other description***
***Age***	> 5 years	Children greater than 5 years are stronger (have higher levels of iron)
		11 to early teens are able to understand MC and care for themselves
***Cost***	Free	Because it is for the prevention of HIV
		Could be barrier to service delivery
		Costs already incurred for transport to health facility
***Who should cut***	Health Worker or Traditional cutter (if experienced)	Health worker because they have medical expertise
		Traditional cutter would need training, but could offer treatment with traditional medicine
***Awareness***	MC program in ESP not well known to women	
**Young men Wewak n=8**		
***Factor***	***Consensus vote***	***Other descriptions***
***Awareness***	For everyone	Radio, television, peer group communication, newspaper, counsellor, general community awareness
		Health and hygiene, to prevent HIV, for custom purposes
***Age***	– 8 years	
***Location***	Range of options	Health facility, *Haus man* or private doctor/traditional cutter
***Treatment***	Combined health facility and traditional (to reduce costs)	Health Facility for initial injections, medication, iodine and dressings
		Traditional treatment for any follow up needs
***Types of cut***	Circumferential cut and Dorsal Slit	Both straight cut (dorsal slit or longitudinal incision only) and round cut (full circumferential cut) should be available
***Access***	People should have choice	Service brought to villages
		Build health facility closer to rural people
***Cutter***	Health worker or traditional cutter	Either male or female
***Cost***	Free	
**Eastern Highland Province workshops**		
**Older women`s group (Village) n=9**		
***Factors***	***Consensus vote***	***Other details***
***Age***	>15years	
***Cost***	Free	
***Location***	In Village	
***Service delivery barriers***	Shame (to go to health facility) and Cost	Cost was seen as most significant barrier
***Who should cut***	Health worker	
***Young women`s group (Village) *n=12**		
***Factors***	***Consensus vote***	***Other details***
***Location***	Aid post	Prefer aid post over hospital due to ease of access
***Age***	>13 years	
***Practitioner***	Health worker who is unknown	
***Cost***	Free	Considered most important issue
***Men`s group (Village) *n=20**		
***Factors***	***Consensus vote***	***Other details***
***Location***	In Village	Build a House in the Community for procedure
***Who should cut***	Local cutters	To feel more comfortable
		Continued employment of local cutter
***Awareness***	For entire community	
***Timing***	Regular service	
**Community leaders (Lay) group Goroka n=7**		
***Factors***	***Consensus vote***	***Other details***
***Awareness***	Explanation of what MC is to everyone	To make informed decision
		Use of community level representative appointed locally to be present in the community at all times, understands local language, to assist in breaking down shame.
		Allowance for rep
***Location***	Aid post	To avoid all complications male circumcision must not be done outside of health facility
	Urban Clinic	Health facilities must be equipped with proper equipment
	Hospital	
***Cutter***	Health Care worker only	Doctors and nurses (must be trained certified medical practitioner)
		Gender Sensitive for older population (i.e. male HW only)
***Age***	>10years	Foreskin easier to cut
		Old enough to care for the treated wound
		Preparation for prevention of HIV before sexual debut
***Cost***	Free	Free of charge for both procedure and medication because
		-It is a government HIV strategy
		-Higher attendance and number of males circumcised
**Specialised community leaders Goroka (health workers, prison officers, department of health) n=11**		
***Factors***	***Consensus vote***	***Other details***
***1. Location/access***	Local service	
***2. Who will cut***	Health worker or local cutter (with training)	Limited human resources available
	Male only	
***3. Age***	> 10 years (before sexually active)	∙Service should be available to a wide range of ages
		∙Concern over consent and compliance
***4. Cost***	Free	∙Incentives for men (bus fare/tea or coffee)
		∙Incentive for local cutter or CHW to recruit
***5. Training***	Relevant to PNG, Supervisor medical officer only	
***6. Compensation***	Potential for legal action due to complications	
	Would impact on community acceptance of program	

##### Awareness

Knowledge of MC within ESP, varied considerably by gender and was a concern for both men and women. For example, despite an adult medical MC program for HIV prevention already established in Wewak, men from Wewak settlement areas explained that although they had heard of the program they still thought raising awareness was an important issue:

*I want more awareness to be done and those men like me who have already been circumcised must go into their communities and inform their friends and relatives so that they can feel that it is good to go and have a complete circumcision [full circumferential cut] done.* Man, focus group discussion, ESP

In contrast, women from ESP who participated in both the participatory workshops in Wewak and FGDs in their communities had very limited understanding both of the MC program in ESP, and of penile cutting practices among men in their communities and also raised the importance of awareness. Conversely, in WNBP, women seemed to have a clear understanding of traditional penile cutting practices within their communities as they played significant roles in the celebrations. Further details are discussed below.

#### West New Britain Province

##### Where should the program be located?

In WNBP, where traditional penile cutting is still widely practiced, there was strong support for male circumcision to be completed by health worker and incorporated within traditional rituals and observances. The location of the ceremony celebration in the *haus boi* held significant cultural meaning to both men and women, and seemed to define and distinguish customary practices. However, men and women were divided about the location of where the procedure should take place in order to align the procedure with traditional culture. Suggestions were made by men that the procedure could be done in a health facility but that all of the other cultural ceremony should be completed in the *haus man*.

*…the government must know, all the men must be circumcised. Like in the health facility or such. The government must know and talk strongly about circumcision. We [my tribe] will also support it however it must go through custom. If the government can acknowledge and strengthen our custom work with something, or it can fund with some money to erect a house for circumcision where we will buy things like blade cutter or such things to put in there. Later, we will go through initiation customarily. All the boys will go into the house for circumcision.* Man, in-depth interview, WNBP*.*

Women in these traditionally circumcising communities acknowledged that the role they played in circumcision rituals was an important part of their meaning and contribution to the community and they were reluctant to consider incorporating a health facility visit into these traditions.

*The government wants to do it in the health facility, however, we the mothers sitting together here have discussed and we want to go about it the customary way. We want to build a “haus boi” here where doctors can come here and cut children’s foreskin here. They have to come here so when they cut them, there will be special mothers who will take care of them. The doctor can come and give injection and such in the “haus boi” which will have custom to it. After six days, we and the doctors who came and cut the children’s foreskin will get togather and make a feast and have food with these children and later they can go. This is what we mothers in this community think. If they want to cut them in the health facility, we will think otherwise. It is like the government has no respect for our custom. It is just cutting the foreskin of men and there is no custom to it because like we said already, circumcision is a big thing. The custom we follow here is that we kill many pigs after they cut children’s foreskin. That is why we have this idea*. Woman, Focus Group Discussion, WNBP

##### Impact of traditional payments on location

The cultural expectation of payments was also suggested to impact on preferred models of service delivery. Some respondents in WNBP felt that obligations surrounding customary circumcision could determine service uptake depending on economic position. For example, preference for a particular location may depend on the wealth of a family in traditional cutting societies. A poor family may prefer to go to a health facility as they then could complete the procedure in secrecy, fulfilling customary requirements in part whilst avoiding the cost of celebrations associated with traditional cutting events. A rich family would be able to afford the cultural celebrations and most likely prefer that the procedure be conducted in the community. As one woman described:

*To cut children in the health facility, this depends on individuals. A man who is poor can go to the health facility, to hide and do it. A man who is rich can do it in the village.* Woman, Focus Group Discussion, WNBP

Engaging in celebrations was a way of consolidating a family’s place within local society, as one informant described:

It’s an experience and an opportunity where ah for one, family prestige that I was able to go. People who don’t have pigs will not go through that process for instance. It shows that my people have been working and can be counted on as a member of the community to supply food and pigs. It is the pride for each family to see their son go through that process and I feel proud that I went through the process. Because it’s a requirement placed on families in the community that boys go through the process, its celebrated and when I reached that stage I went to an adulthood stage where you know they slaughter pigs and this time with big* people like me. Man, In-depth Interview, WNBP

(*“Big Man” is a term commonly used to indicate a title of status or leadership)*.*

### Diversity in communities where traditional forms of penile cutting are not carried out

#### Guaranteeing confidentiality and discretion

Issues of guaranteeing confidentiality and discretion for young men were raised in traditionally cutting communities, however, this issue seemed more prevalent in urban areas and communities where traditional penile cutting was not a part of their cultural backgrounds. In the non-traditionally cutting communities and urban areas of NCD and EHP, respondents reported that the program would need to address the reluctance of men, particularly youth, accessing the health system via the usual channels. Suggestions provided for this reluctance included the young men’s general reserve about discussing matters pertaining to sexual health; potential discrimination at formal health settings due to cultural background or practices; anticipated attitudes of health workers (HWs) towards the practice; economic burden in accessing a health facility; or the risk of looking weak in front of peers.

*Sometimes, if the small boys hear that the men are removing their penis foreskin, they will make fun and call them “Papa Kela”* (‘bald man’ in tok pisin). Young Women, Focus Group Discussion, NCD

Participants raised a number of considerations that needed to be overcome to accommodate issues of shame and reluctance to participate in a program based in a formal health setting. To accommodate the need for discretion some considered that major hospitals would actually increase secrecy for the young men whilst others suggested that hospitals were too public and that many boys would be reluctant to attend, and therefore resort to doing it themselves. Others advised that the procedure should be completed at community level, but in secrecy; for example, doctors should attend villages upon appointment:

*I think it’s good to come to the village and do circumcision because many men in the village are embarrassed to go to the health facility*. Young man, Focus Group Discussion, EHP

#### Who should do the cutting?

Issues around privacy and familiarity of the HW also dictated preferred service provider with many suggesting that it should be someone who was unknown to the patient. For a few, the preference was for a male HW due to potential issues of embarrassment for the patient. One young man also described that issue of embarrassment may also extend to a female practitioner:

*I think it will be good if a male doctor cuts it because if a female doctor cuts it, the patient will be embarrassed. When afraid she* [the female doctor] *might make a wrong cut.* Young man, focus group discussion, EHP

Having a man perform the procedure would also guarantee secrecy according to some:

*The government must set it in a centre where only the males know. Only the males will know and the staff too must get the mature men’s’ opinion bit by bit and then will advise them and say this is this so will bring them to a session, secretly circumcising them*. Young man, Focus Group Discussion, EHP

For others, issues over cultural expectation would need to be accommodated and would dictate who would be able to perform the procedure:

*Well of course, guys or boys they would feel comfortable because in some cultures males are dominant so if a female touches it [penis], its disrespect you know.* Man, focus group discussion, NCD

Men and women differed in their opinion as to whether a HW or a local cutter should carry out the procedure. In EHP communities, women felt that a HW from outside the community who was unknown to them would be most appropriate, in order to prevent embarrassment and improve service uptake (Table 3). In contrast, men preferred a local cutter, and highlighted the potential loss of income if HWs exclusively delivered this service. The training of local cutters was also supported by key stakeholders and HWs who felt that frontline health staff were already under pressure and the use of local cutters could alleviate some of their work burden.

#### Age and consent

In EHP, there was also much debate about the age of the child and the need to obtain informed consent. Some participants felt MC should be carried out before sexual debut:

*They must start circumcising from 3 years old upwards because these days, young children watch a lot of movies and they see men and women sleeping together in the movies and their brains are matured.* Young man focus group discussion, EHP

Circumcision of younger children was also a preference for men in NCD with some indicating that babies should be considered.

*The Europeans do it when the baby is born, maybe one year old or two year old or six months or something like that… so maybe it’s good for us Papua New Guineans to stand up and do like that too in our country and community.* Man, focus group discussion, NCD

Women in both EHP and NCD, however, suggested the target age group should be much older. For example women from a village in EHP suggested that MC be offered to youth aged 13 years and above (Table 3). Women reasoned that an older age would mean that the boy was stronger physically and mentally to withstand the procedure. Women in NCD advised that MC should be carried out on boys aged 15 – 20 years because younger than that, they would have fear of pain.

A common concern amongst all participants in EHP and NCD was consent, and particularly how this would dictate an appropriate target age group for the intervention. For example, consent and compliance was raised as an issue in EHP during the participatory workshops. However, participants were divided over whether it was ethical for parents to provide consent for MC for younger children (<10years), suggesting that it would be better to wait until the boys could make a choice and provide consent themselves. Others felt if the target age were raised to >13 years and boys were allowed to make the choice themselves this would impact on uptake because boys would be more likely to decide not to have the procedure. This lead to intense debate about the priority for a HIV prevention program and the need to ensure that boys would be ethically encourage to come for MC. This issue of choice was a common concern amongst a number of participants

*I believe that people should be given the choice I mean whether or not to get circumcised. Because although circumcision comes out as a prevention method, it should not be forced on to other people because different people have different believes and their rights have to be respected.* Man, Focus Group Discussion, NCD

### Issues of commonality amongst all participants

A number of common themes were identified amongst both traditionally cutting and non-traditionally cutting communities including flexibility of type of cut offered, cost, awareness and, community collaboration. For type of cut, for example, despite uncertainty around the protective efficacy of the dorsal slit (longitudinal cut of foreskin only) for HIV prevention, both traditionally circumcising communities and traditionally non-circumcising communities reported preference for it, with many participants suggesting that both the dorsal slit and full circumferential cut options be made available.

Cost was also a key consideration and of concern to all participants, with the consensus that MC should be provided for free in any future service. For traditionally circumcising communities, differentiation was made between cost involved as part of traditional celebrations (which was considered integral to the practices) and costs incurred due to fees involved in accessing a health program.

*I want this service to be free. When it’s free all the men will be happy to go.* Young man in-depth interview, EHP

Some argued that because it was being encouraged by the government for the prevention of HIV, then the onus of payment should be on the government.

*I would expect the government to carry out this circumcision for free because the government should be the main people trying to stop the expansion of HIV and AIDS.* Man, focus group discussion, NCD

Participants also raised concerns about cost as a potential barrier to service uptake if not considered thoughtfully. For example, cost of the service and the associated costs of access to services were identified as significant barriers, particularly in remote communities.

*…it won’t be good if you do it in towns only. This operation should be done in rural areas… People in the village find it hard to come into town because it is costly for them.* Man, in-depth interview, ESP

Even in urban settings, men acknowledged that unemployment was prevalent and that most young men would not readily have access to cash. The convenience of performing the procedure outside a health facility was a more desirable option for a few respondents, as one man who had a penile cut outside a health facility explained:

*…going to the health facility* [to get cut] *is expensive…one, it’s going to take time to circumcise. Two there is pay,* [fee] *and it’s very expensive….* Man, In-depth Interview NCD

#### Ensuring community collaboration

The highest priority identified by all participants in the in-depth interviews, workshops and focus group discussions for a successful future MC program was the need for community awareness. Respondents emphasized the need for clear communication about the MC program to everyone. In particular it was considered that women must be well informed about the advantages and disadvantages of MC as they will be primarily responsible for providing consent for their children to undergo the procedure.

*Regarding the awareness, when they make awareness, they must tell the women too regarding circumcision.* Young man, focus group discussion, EHP

The importance of fostering good community relationships was seen of upmost importance with a need to develop a strong relationship with the community, share food and create mutual respect, essential for program success. This would allow the community to feel more a part of and at ease with what was happening. The person chosen to deliver the awareness would also be integral to the success of the program. As one young man from EHP explained:

*Village people don’t trust just anybody that comes and talks. They trust their own squad, one age group. So for this sort of education, when a villager gives it* [education] *to his kind, they will understand it. An educated person giving it [education] to an educated person they will understand it.* Young Man, Focus Group Discussion, EHP

## Discussion

In exploring options for the implementation of a MC program for HIV prevention in PNG this study has highlighted the conflicts between health system practicalities and realities, and community expectations of how such programs should be delivered. Further to this, implementation of a health program that has parallels with prevailing traditional and contemporary practices which are grounded in diverse socio-cultural contexts are likely to increase tensions if not addressed in an appropriate way. Suggested service delivery barriers and facilitators were also diverse and included issues of shame, appropriate location in which to conduct the procedure, who should be involved and how, and who the target population should be. Conversely, awareness, affordability and ease of access were consistently identified as important contributors to meeting community needs and if not addressed will likely increase risk of program failure. Attitudes and perceptions towards circumcision and how services should be delivered will influence uptake and community participation approaches provide an option for exploring these challenges.

### Working against the grain

Health programs are underpinned by the interests and expectations of the health system that cannot be down played and this needs to be acknowledged when according true community participation [1,39,42]. Community perception of health service delivery often does not recognise the same boundaries of the health system, and tensions may exist with medico-legal requirements and bio-medical best practice. For example, potential for misperception has been highlighted in introducing MC for HIV prevention in some communities in South Africa due to conflicting messages of abstinence and sexual responsibility evident in the health program compared to the sexual privilege and masculine virility messages that accompany traditional circumcision and initiation ceremonies within the community [75-77]. In our study, there was clear evidence that communities in PNG endorsed forms of penile cutting that have not been confirmed to provide protection against HIV acquisition. These findings reflect earlier research by our group which demonstrated that many men opt to perform penile cutting within the community without a medically trained practitioner [9,17]. Concerns about access, cost and reluctance of men to access formal health services for MC also resulted in some mixed proposals for risk mitigation, with some strategies beyond the capacity or legal framework of the health system (such as providing equipment to perform cutting at home without attention to counselling on risk reduction and post-operative care). Complex interconnectedness between policies, health system capacity, legislation and system hierarchies form an invisible and subconscious web of forces with socially constructed scientific knowledge, established practices and diverse community voices. If not adequately addressed, the competing priorities and agendas of the key stakeholders pull program intentions in many directions `working against the grain` with varying results, as tears and strains in relationships are restored, loosened or pulled further apart. Identifying this conflict is one step, understanding how to involve communities in meaningful and sustainable ways to develop a culturally safe, assessable and affordable program is integral to success.

### The role of participatory approaches

Community engagement and participation has played a critical role in successful communicable disease control and elimination campaigns in many countries [2,57,60]. In HIV prevention programs in particular, community participation has shown significant community-level change in safe sex and at-risk drug behaviours following health promotion programs that employed community coalitions [47,78]. Community-centred approaches have also been identified as integral to working in resource-limited settings, where at-risk populations are often marginalised within the broader community and considered vulnerable both in terms of increased risk of HIV and sexually transmitted infections (STIs), and due to stigma, poverty, social exclusion and lack of access to education, healthcare and other essential services [57,79,80]. Collaboration with community members should aim to place the whole person, and not just the disease, at the centre of health interventions [1,39]. This empowers people to assume responsibility for their own health, and facilitates effective partnerships to be built between the health program and the community [49,81]. The design and success of community participation may however vary significantly due to geographic location, disease impact, socio-cultural environments, political context, economic conditions, resource availability and health policy [2].

Successful involvement of communities in meaningful and sustainable ways in health services requires a robust understanding of local approaches for community involvement in the face of political, social, economic, and epidemiological pressures [2,36,39,42,82]. This research shows that key to developing program models of service delivery was appreciation of the difference in expectations and traditions of different cultural groups in PNG. All respondents agreed that different areas would indeed respond better to tailored service delivery of MC programs designed specifically for their communities. Many studies have shown that even in areas where from an external point of view, modern and indigenous practices conflict, local people find ways of making their own cognitive adjustments and of integrating their need to resort to both traditional and modern options, with positive and negative results [39,83,84]. Engagement in community collaborations and community participation in the design and implementation of health programs allows for increased ownership as well as avenues to address emerging concerns or questions. Community participation should be an evolving process, whose tensions and contradictions are created and resolved through stages of demands and accommodations [41,42,85,86].

To assist with building partnerships and trust in the implementation of any future MC program, community members and stakeholders specified that awareness and education be high on the program agenda particularly for women. This was also similar to some studies in Africa where education and awareness of female partners or mothers in the uptake of MC was considered important [16,87-89]. Awareness is knowledge empowerment that includes clear understanding of health service purpose as well as key aspects of program requirements. The existence of secular trends in the community driven by increased communication of MC trial results, the discussion about MC services and an increasing appreciation of the distinction between MC for health reasons and MC as a traditional rite has also been described as a potential issue in studies in Africa [90-92]. There is therefore a responsibility of the health system to acknowledge internal and external influences on awareness and management of health programs, particularly if there is a potential for misperceptions to develop. Participatory approaches allow for collaboration with those affected by the issue being studied for the purposes of education and taking action or effecting social change [93].

### Engaging traditionally circumcising communities

Most often, communities who engaged in traditional cutting in Africa were largely opposed to the medically performed procedure due to issues related to stigma, cost and cultural significance [5,75,94,95]. Although maintaining cultural significance plays an important part in PNG, many respondents from traditionally circumcising communities were supportive of a HW completing the procedure as part of traditional ceremony in the village. In PNG, location and people involved seem to define meaning rather than the procedure for traditionally circumcising communities. The interest in allowing biomedical HIV prevention strategies to be integrated with traditional practices offers significant opportunity to mitigate potential complications resulting from procedures performed by non-health workers (a goal of the health system); and to involve and honour the symbolic roles of traditional leaders and circumcisers to provide traditional legitimacy (goal of the community) [5,75,94]. However, as has already been recognised in some African settings, MC performed in a clinically controlled setting, even if it is endorsed by traditional and contemporary communities, is likely to be hampered by health system capacity in resource-constrained settings such as PNG [96]. Setting realistic and achievable goals during initial collaborations are essential to avoid unrealistic expectations of the program, to increase trust and to prevent dissonance between policy makers, health workers and communities.

The use of a traditional cutter was also suggested and often preferred and may assist in alleviating human resources burden in the health sector of PNG. Lessons of integration of traditional cutters may be learnt from the use of traditional birth attendants (TBAs) in maternal health. TBAs share similar characteristics to traditional penile cutters in that they generally lack formal medical training, but have been playing their role in communities for generations [97]. Competence, confidence and connectedness to the health system are key factors identified in the understanding of TBA roles and their potential for success [97,98]. Traditional cutters have advantages in that they live within the community, understand local culture and customs and are likely to be well respected by community-members thus increasing acceptability and uptake of interventions and galvanising behaviour change [99]. However, without the proper training and supervision, these providers may be firmly entrenched in traditional customs that may be either potentially harmful or delay the receipt of appropriate care [100,101]. Expectation placed onTBAs also need to be realised such as the inability of TBAs to reduce maternal mortality [102]. Also, as has been learned from successful TBA training and support programs, partnerships between traditional cutters and the formal health system would need to be strong and would require frequent and quality interactions between supervising health worker and traditional cutter.

### Recommendations and implications for program planning and implementation

The sheer socio-cultural and linguistic diversity of PNG demands that health services be astute to local needs. A complex intervention such as MC for HIV prevention that already holds diverse socio-cultural meanings in PNG also confirms that one size will not fit all and this is likely to be the case for other health interventions delivered in this context. Decentralised health services need to have the capacity and competency to contextualise the way any health program should be delivered locally in negotiation with the community as equal partners, whilst ensuring efficacy and quality. Expectations that health planners and donors may place on rate and size of scale up may be unrealistic if these key local considerations are not realised or incorporated into effective and robust community partnerships. Service provision plans must be highly flexible to respond to changes in the needs and expectations of communities. There is also need for health program planners to continually engage in self-reflection and dialogue to understand the partnerships developing between the community and the program. Program integration within the cultural fabric of communities will not only assist in strengthening the health system in general, but also community relations for future health programs, if done in a community-centred way.

### Limitations of Study

As is the nature of qualitative research, our findings are limited in their ability to be generalized to the wider population. This research was however conducted in four distinct regions of PNG and provides insights into the complexities of future service delivery of MC for HIV prevention in PNG. Another potential limitation of the study is the possible loss of nuances that can occur through the direct translation from ‘*tok pisin*’ to English of the FGD and IDIs recordings by the local research officers. Care was made to ensure that meanings were reflected in the translations through re-checking and confirming of information with researchers, and review of field notes as necessary.

## Conclusions

Tensions exist in the design and implementation of health programs however, identification of facilitators and barriers through meaningful collaborations with communities will assist in developing safe and culturally appropriate health programs. Building trustful partnerships with communities are critical and are the result not only of history, culture and tradition, but also the application of participatory community engagement in health programs. It is also important that women be included in awareness campaigns about MC with the aim to empower greater knowledge about sexual health in general. Addressing these issues are essential to develop programs that will accommodate the presence or absence of practices that form the socio-cultural fabric of communities.

## Abbreviations

EHP: Eastern Highlands province; ESP: East Sepik Province; FGD: Focus group discussion; HW: Health worker; IDI: In-depth interview; MC: Male circumcision; NCD: National Capital District; PNG: Papua New Guinea; WNBP: West New Britain Province.

## Competing interests

The authors declare that they have no competing interests.

## Authors’ contributions

AT, AV and PSH participated in the conception of study design with feedback and guidance from AK. The field research activities were supported by AK, MK, HA and RN during stage 1 and AT, AV, and RN during stage 2. Data analysis was initially carried out by AK, MK, HA, RN and AV during stage 1. In stage 2 AT re-analysed all data with support from PSH, AV and AK. The Manuscript was drafted by AT, PSH, AK and AV with contributions from MK, HA, JK, PS. All authors read and approved the final manuscript.

## Pre-publication history

The pre-publication history for this paper can be accessed here:

http://www.biomedcentral.com/1471-2458/13/749/prepub
